# UV-activated ZnO films on a flexible substrate for room temperature O_2_ and H_2_O sensing

**DOI:** 10.1038/s41598-017-05265-5

**Published:** 2017-07-20

**Authors:** Christopher B. Jacobs, Artem B. Maksov, Eric S. Muckley, Liam Collins, Masoud Mahjouri-Samani, Anton Ievlev, Christopher M. Rouleau, Ji-Won Moon, David E. Graham, Bobby G. Sumpter, Ilia N. Ivanov

**Affiliations:** 1 Center for Nanophase Materials Science and Institute for Functional Imaging of Materials, Oak Ridge National Laboratory, PO Box 2008, Oak Ridge, TN USA; 2 The Bredesen Center for Interdisciplinary Research and Graduate Education, 444 Greve Hall, 821 Volunteer Boulevard, Knoxville, Tennessee United States; 3 Microbial Ecology & Physiology Group, Biosciences Division, Oak Ridge National Laboratory (ORNL), PO Box 2008, Oak Ridge, TN USA; 4 Computer Science & Mathematics Division, Oak Ridge National Laboratory, PO Box 2008, Oak Ridge, TN USA

## Abstract

We demonstrate that UV-light activation of polycrystalline ZnO films on flexible polyimide (Kapton) substrates can be used to detect and differentiate between environmental changes in oxygen and water vapor. The in-plane resistive and impedance properties of ZnO films, fabricated from bacteria-derived ZnS nanoparticles, exhibit unique resistive and capacitive responses to changes in O_2_ and H_2_O. We propose that the distinctive responses to O_2_ and H_2_O adsorption on ZnO could be utilized to statistically discriminate between the two analytes. Molecular dynamic simulations (MD) of O_2_ and H_2_O adsorption energy on ZnO surfaces were performed using the large-scale Atomic/Molecular Massively Parallel Simulator (LAMMPS) with a reactive force-field (ReaxFF). These simulations suggest that the adsorption mechanisms differ for O_2_ and H_2_O adsorption on ZnO, and are governed by the surface termination and the extent of surface hydroxylation. Electrical response measurements, using DC resistance, AC impedance spectroscopy, and Kelvin Probe Force Microscopy (KPFM), demonstrate differences in response to O_2_ and H_2_O, confirming that different adsorption mechanisms are involved. Statistical and machine learning approaches were applied to demonstrate that by integrating the electrical and kinetic responses the flexible ZnO sensor can be used for detection and discrimination between O_2_ and H_2_O at low temperature.

## Introduction

Zinc oxide (ZnO) has electromechanical and photocatalytic properties that have been used in a wide range of applications, including piezoelectric and photocatalytic devices, transparent conductive electrodes for solar cells, and environmental gas sensors^1–5^. For example, Shaban used Na-doped ZnO nanostructures for enhanced CO_2_ detection, and Saraswathi demonstrated that DC magnetron sputtered films of polycrystalline ZnO can be used for highly sensitive humidity sensors^6, 7^. Although the environmental effects on the electrical properties of ZnO films are well studied, the response mechanisms are still not fully understood. The resistive and capacitive responses of ZnO films to gases typically require operational temperatures above 350 °C to provide sufficient energy to ionize the surface oxygen atoms, and promote adsorbate desorption, and thus improve recovery and sensitivity of the sensor. However, high temperatures are not compatible with flexible polymer substrates, can lead to grain growth and long-term drift problems in ZnO films. UV irradiation of ZnO films has been used as a viable alternative to activate the surface and induce photo-catalytic analyte desorption^8–13^.

The resistive response of an n-type ZnO film relies on the interaction between oxygen vacancies at the ZnO surface and charge accepting (or donating) adsorbate molecules. O_2_ and H_2_O both compete for the same adsorption sites on ZnO surfaces; however, the adsorption mechanisms differ. Under low thermal activation (T < 100 °C) molecular oxygen (O_2_) physisorbs to the ZnO surface resulting in minimal resistance response, since O_2_ is not an efficient electron trap. At temperatures exceeding 350 °C, however, O_2_ is ionized to form efficient surface electron traps, such as O_2_^−^ and O^−^, which diminishes the thickness of the conduction layer (grain boundary), increases the thickness of the depletion layer (grain), and consequently results in increased resistance^1, 2, 14^. On the other hand, at low temperatures water adsorbs to the ZnO surface in molecular form (H_2_O) via hydrogen bonding or physisorption, while at higher temperatures water chemisorbs to the ZnO surface through an acid/base reaction at a Lewis base site. Chemisorbed water forms a surface hydroxyl (-OH) functionality, leaving an isolated proton to react with lattice oxygen and form another surface hydroxyl^15–17^. Adsorbed oxygen and water species have large dissociation energies, resulting in negligible desorption and poor reversibility of sensor response at low temperature^18, 19^.

**Figure 1:**
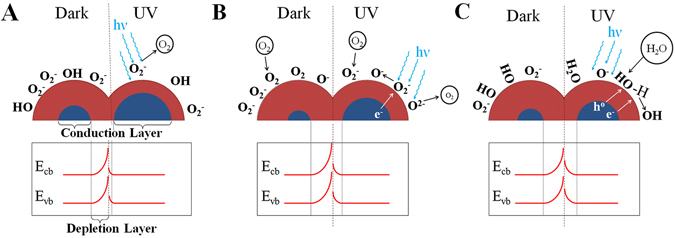
Schematics of the conduction (grain) and depletion (grain boundary) regions of ZnO illustrates the changes in valence (E_vb_) and conduction (E_cb_) band structure of ZnO nanoparticles between dark and UV conditions while under vacuum (**A**), with exposure to oxygen gas (**B**), and with exposure to water vapor (**C**).

Irradiation of ZnO by photons with an energy greater than the band gap (3.37 eV) results in the formation of electron-hole pairs in the depletion region. Under vacuum, the photogenerated-electron induces desorption of surface oxygen species, and decreasing the energy barrier between ZnO grains, leading to lower film resistance, Fig. 1A ^20, 21^. Oxygen molecules can be ionized by the photo-generated electrons, resulting in the chemisorption of ionic oxygen to the ZnO surface (O_2_ + *e* ^−^_(*hv*)_ → 2 O^−^_ads_), leaving photo-induced holes in the depletion layer, thereby increasing the film resistivity^22, 23^. The photo-induced holes can react with chemisorbed oxygen species, leading to molecular O_2_ desorption from the ZnO surface. The process leaves an excess of electrons in the conduction band, diminishes the energy of the depletion layer, thus decreasing ZnO film resistivity, Fig. 1B ^13, 24^.

The adsorption of water vapor at the ZnO surface displaces adsorbed atomic and molecular oxygen, releasing electrons into the depletion layer, and resulting in decreased resistivity^14^. Under UV irradiation, isolated H_2_O molecules are physisorbed on the ZnO surface without dissociation and hydrogen bonding of successively adsorbed H_2_O molecules induces dissociation of adjacent water molecules, removing both photogenerated electrons and holes, Fig. 1C ^25, 26^. Since interaction of O_2_ and H_2_O with ZnO proceeds via different mechanisms, it is expected to lead show observable difference in ZnO electrical response at room temperature. However, it is not typically possible to differentiate between O_2_ and H_2_O response from the electrical resistance response alone, regardless of whether thermal- or UV-activation is implemented.

Here we report on an approach to differentiate between O_2_ and H_2_O responses using a combination of statistical methods and machine learning techniques applied to impedance responses of ZnO films under dark and UV-irradiation at room-temperature. Our molecular dynamic (MD) simulations show that the adsorption energy on ZnO differs for O_2_ and H_2_O, and that the adsorption energy is ultimately influenced by the extent of ZnO hydroxylation, as well as the presence of Zn and O vacancies. We argue that by integrating resistive, capacitive, and time-dependent sensor responses with statistical analysis, it is possible to discriminate between analytes which interact with the sensor material via similar, but distinct, mechanisms. Kelvin Probe Force Microscopy (KPFM) measurements confirm that confirm that the adsorption of water on the ZnO surface leads to decrease in surface potential, due to adsorbed water molecules increasing the surface electron density.

## Results and Discussion

**Figure 2:**
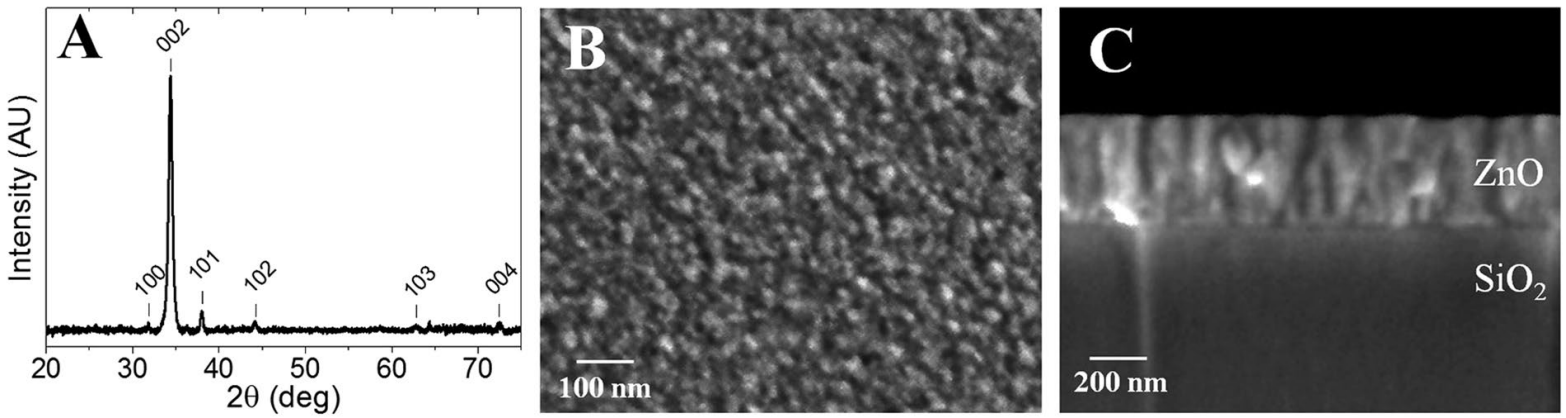
X-ray diffraction spectra showing the c-axis oriented hexagonal structure of the ZnO film on a polyimide substrate (**A**). Surface (**B**) and cross-section (**C**) SEM images indicated a film of 85 nm ZnO film deposited by PLD on a SiO_2_/Si substrate at 250 °C.

Pulsed-laser deposition (PLD) of ZnO was found to form a polycrystalline film comprised of c-axis oriented hexagonal ZnO, Fig. 2A. The morphology and crystalline structure of the ZnO films were found to be similar on both polyimide and silicon substrates, substantiating previous reports, thus we focused our efforts on the flexible polyimide substrates^27, 28^. SEM images show 85 nm tall (film thickness), ZnO columnar structures with diameter ranging from ~5 to ~25 nm, Fig. 2B,C. The electrical properties of ZnO film, and can be modeled as the sum of the contributions of ZnO grains and the grain boundaries, Fig. 1.

**Figure 3:**
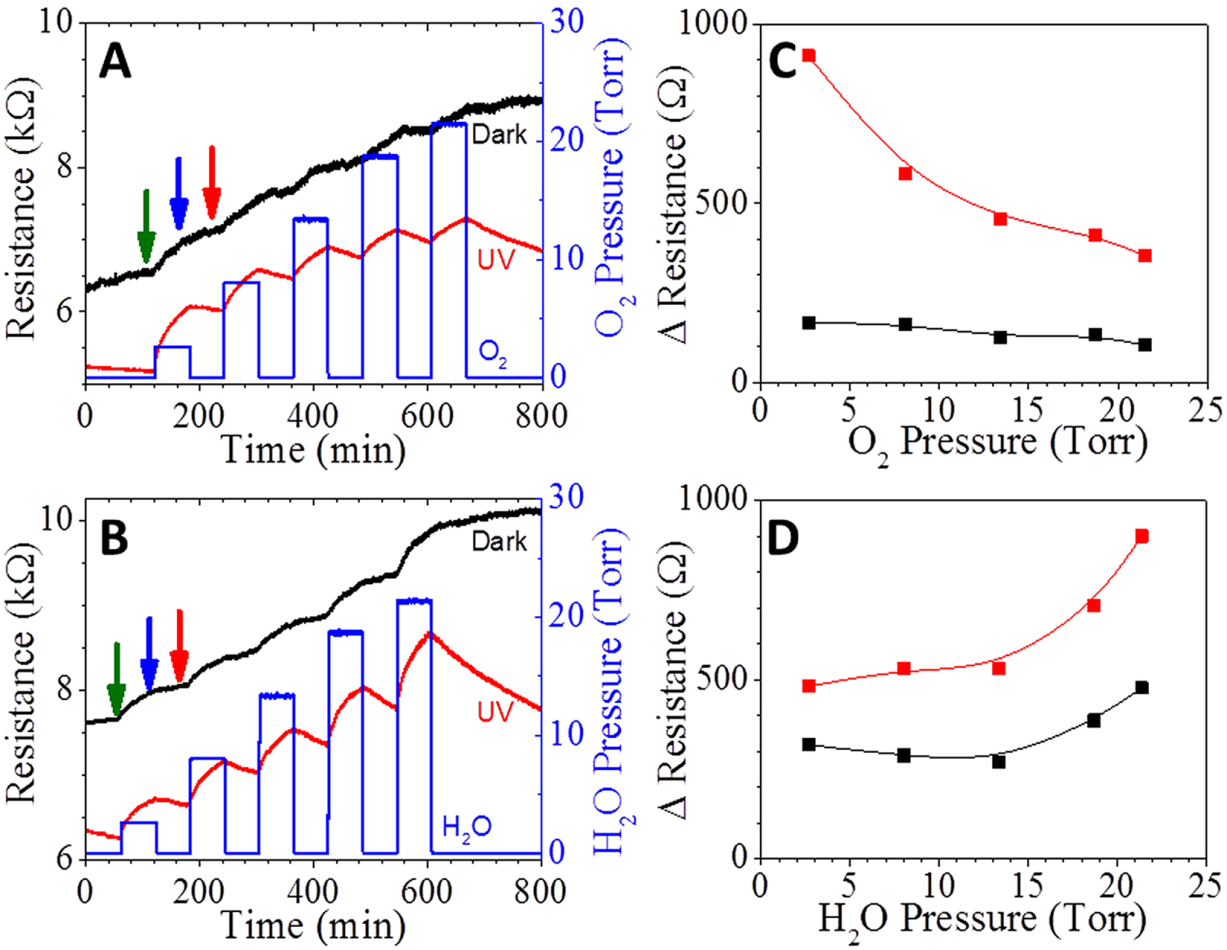
Resistive response of ZnO films, in dark (black) and UV (Red) conditions, to pulses of increasing pressures of O_2_ (**A**) and H_2_O vapor (**B**). The pressure of analyte pulses are depicted by blue traces. Green, blue and red arrows represent the point where full impedance spectrum were measured (shown in Fig. 4). Relative resistance responses to O_2_ (**C**) and H_2_O (**D**) pressure pulses, referenced to vacuum, were determined from the change in resistance from immediately before the pulse to the end of the pulse. UV Irradiation has an exponential effect on the O_2_ trend, while irradiation did not appear to change the H_2_O trend, but simply increased the resistive response. The decrease in response to increasing O_2_ partial pressure is similar to the response of consecutive pulses of a single O_2_ partial pressures (Figure S2), suggesting that it may be related to deoxygenation of the film during the UV cleaning process, prior to measurements.

The ZnO films were exposure to controlled pulses of oxygen gas and water vapor at ambient temperatures while monitoring in plane resistance and impedance response of the films. A series of 60 minute pulses of increasing oxygen pressure and water vapor were introduced into an environmental vacuum chamber (response period). The sample was exposed to vacuum (at 10^−6^ Torr) for 60 minutes following each pulse (recovery period). The experiments were then repeated under UV irradiation. Figure 3A,B shows that exposure to oxygen or water increased the film resistance, and that UV irradiation leads to partial recovery of the resistance. The resistance response as a function of O_2_ or H_2_O pressure is shown in Fig. 3C,D.

Resistance response was found to be proportional to H_2_O-pressure, for pulses exceeding 14.3 Torr H_2_O, and it was about 2 times larger for UV-irradiated film compared to response in dark. At pressure pulses <14 Torr H_2_O the resistance response was negligible. The resistance was partially recovered in the vacuum under UV irradiation, Fig. 3B. The partial recovery of resistance under UV irradiation could be a result of slow photo-induced desorption reactions which requires much longer recovery time to complete the photo-induced desorption reactions of surface oxygen species.

Under UV irradiation the resistance of the ZnO film decreased by a factor of two across the measured range of O_2_ pressures, whereas the resistance decreased slightly without light. This trend challenges previous reports of the resistance increasing upon oxygen adsorption under UV irradiation. The process of photo induced deoxygenation of the ZnO surface under UV irradiation in the vacuum could lead to oxygen deficient surface^22, 23, 29^. Each consecutive pulse of oxygen partially regenerates the oxygen associated with the film, the portion of restored oxygen would be removed during the recovery period under continuous UV-irradiation in vacuum. In such a case, the response of successive pulses is expected to decrease exponentially, until the surface of ZnO is fully oxygenated. To verify this assertion, the ZnO film was exposed to six consecutive, two minute pulses of 0.75% O_2_ and 2% H_2_O. This confirms that UV irradiation in vacuum depletes oxygen at the ZnO surface, and the observed response is a result of the partial recovery of oxygen vacancies in the ZnO film, Figure S2. Our calculations, discussed below, predict that the energy of O_2_ adsorption on a surface with O-vacancies is 0.2 eV higher compared to a clean surface.

The resistance of the ZnO film increased during exposure to either O_2_ or H_2_O, so the resistance response alone does not allow differentiation between the two analytes directly. The observed response and recovery time ae associate with the dynamics and reversibility of the interaction between analytes and ZnO surface were estimated by fitting data with an exponential decay, Table S1. The sensor response and recovery time for H_2_O and O_2_ were found to be independent of the analyte or its partial pressure under dark conditions. Conversely, under UV irradiation the recovery time constant was found to be shorter while recovery time longer, for both O_2_ and H_2_O, compared to dark conditions. Under dark or UV conditions, a time-dependent drift in resistance was observed, even under bias of 100 mV across the ZnO. The possible mechanism behind this phenomenon is electric field-induced ion migration, and the aggregation of ionized adsorbates toward the anode or cathode, and from photocatalytic desorption of oxygen from the ZnO lattice^30, 31^.

Impedance spectroscopy was used to gain a better understanding of the frequency dependent response mechanisms of O_2_ and H_2_O adsorption in dark conditions and under UV irradiation, in the frequency range from 10 Hz to 8 MHz. The impedance responses of ZnO films were measured at the end of each O_2_ or H_2_O pulse, as depicted by color-coded arrows in Fig. 3. The frequency dependent impedance spectra, Figure S3, show that complex impedance and phase shift are sensitive to oxygen and water in the frequencies between 1.5 kHz and 500 kHz, with resistive-like behavior below 40 kHz, and an optimal capacitive response at 150 kHz.

**Figure 4:**
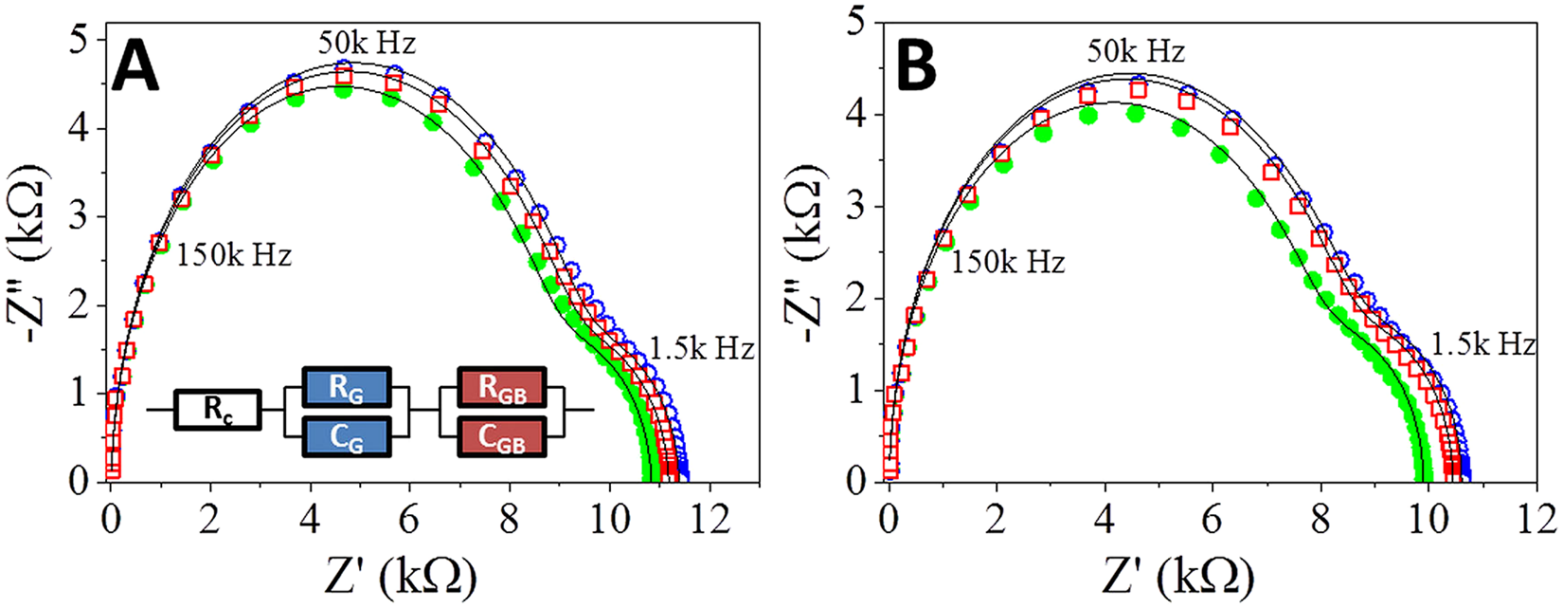
Impedance spectra represented as Nyquist plots of ZnO films measured during O_2_ (**A**), and H_2_O (**B**) pulse experiments under UV irradiation. The solid green points were measured during the initial vacuum, open blue circles during the first pulse (1.7% O_2_ (**A**) or 11.25% H_2_O), and open red squares during the recovery vacuum after the pulse. The solid lines model the data using the equivalent circuit inset. The corresponding colored arrows in Fig. 3 indicate the time at which each trace was measured.

**Figure 5:**
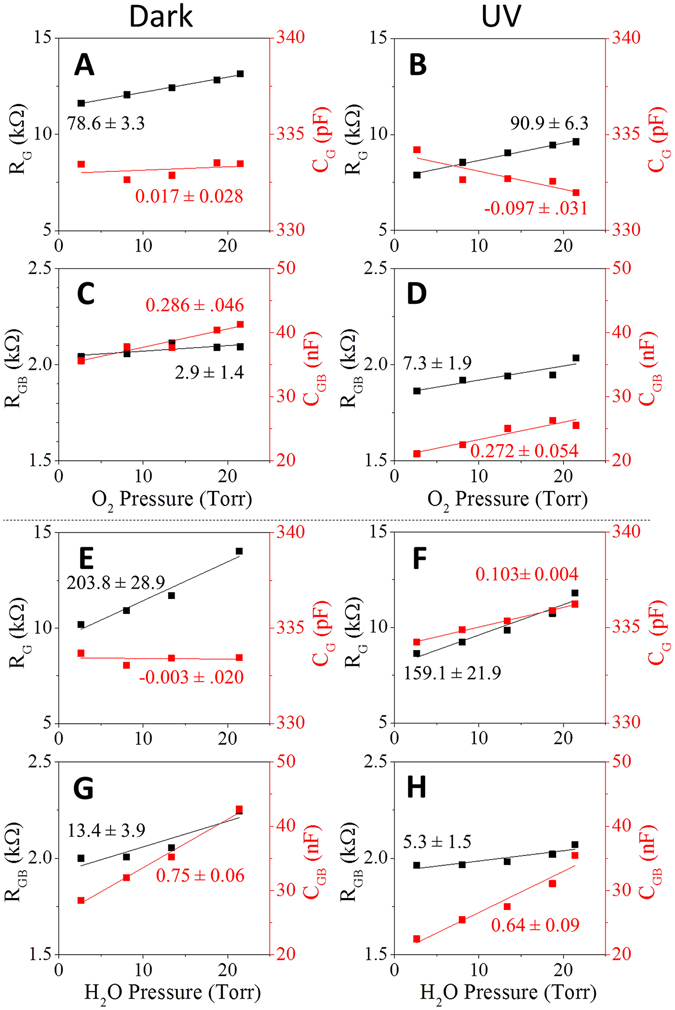
Contributions of the response to pressure from the grain resistance (R_G_, black) and grain capacitance (C_G_, red) to O_2_ (**A**,**B**) and to H_2_O (**E**,**F**); and from the grain boundary resistance (R_GB_, black) and grain boundary capacitance (C_GB_, Red) to O_2_ (**C**,**D**) and H_2_O (**G**,**H**) in dark conditions and under UV irradiation, indicate that the R_G_ and C_GB_ slopes *exceed* the R_GB_ and C_G_ slopes in all cases – i.e., the resistive and capacitive responses are dominated by the grain resistance and boundary capacitance.

Nyquist representation of the impedance spectra measured during vacuum, response, and recovery stages display spectra comprised of two overlapping semi-circles with different time constants, Fig. 4. The high-frequency part of the Nyquist plot (low impedance semi-circle) was assigned to charge transport through the ZnO grains (R_G_, C_G_), while the low-frequency component (high impedance semi-circle) was assigned to charge transport through the grain boundary (R_GB_, C_GB_)^32, 33^. The Nyquist representations of the impedance spectra were modeled using an equivalent circuit consisting of a contact resistance component (R_c_) in series with two sets of a parallel RC components, R_G_C_G_ and R_GB_C_GB_, corresponding to charge transport through the grain and the grain boundaries, respectively, Fig. 4A-inset^34–37^. The values of the RC parameters determined from equivalent model fitting, were plotted as a function of analyte partial pressure, Fig. 5. The sensitivity of each RC component to changes in O_2_ or H_2_O was determined by the slope of a linear fit. Under UV irradiation, the resistive and capacitive responses to O_2_ and H_2_O were found to be defined by changes in the values of R_G_ and C_GB_. The increase in the values of R_G_ and C_GB_ with increasing O_2_ pressure (characterized by slopes of 90.9 Ω/Torr O_2_ and 0.3 nF/Torr, respectively) can be attributed to the formation of ionic oxygen species by reaction between adsorbed O_2_ and photogenerated electrons, consistent with previous reports^14^. The response contribution of R_G_ and C_GB_ was found to be greater for H_2_O than for O_2_ (204 Ω/Torr H_2_O and 0.8 nF/Torr H_2_O, respectively), which is likely due to the dissociative reaction of H_2_O with both photogenerated electrons and holes, as previously noted by Meyer and Dulub^25, 38^. Under dark conditions, C_G_ is independent of O_2_ and H_2_O pressure, while under UV irradiation C_G_ becomes pressure dependent, with slopes of −0.09 pF/Torr O_2_ and 0.6 pF/Torr H_2_O, indicating that the dielectric constant decreases with O_2_ adsorption and increases with H_2_O adsorption. This complex behavior may stem from changes in the surface polarization due to formation of polar species such as ionic oxygen and dissociated water^39^.

The interaction of H_2_O with oxygen vacancies is expected to result in a decrease in surface potential, whereas hydrogen bonding of water at the surface would be expected to result in little surface potential change^25, 40^. Su *et al*. showed that the formation of oxygen vacancies at the ZnO surface alters the work function of the film, leading to an increase in the surface potential^40^. Thus, the adsorption of H_2_O or O_2_ on ZnO is also expected to modify the surface potential due to changes of the surface termination of the ZnO surface^41^.

**Figure 6:**
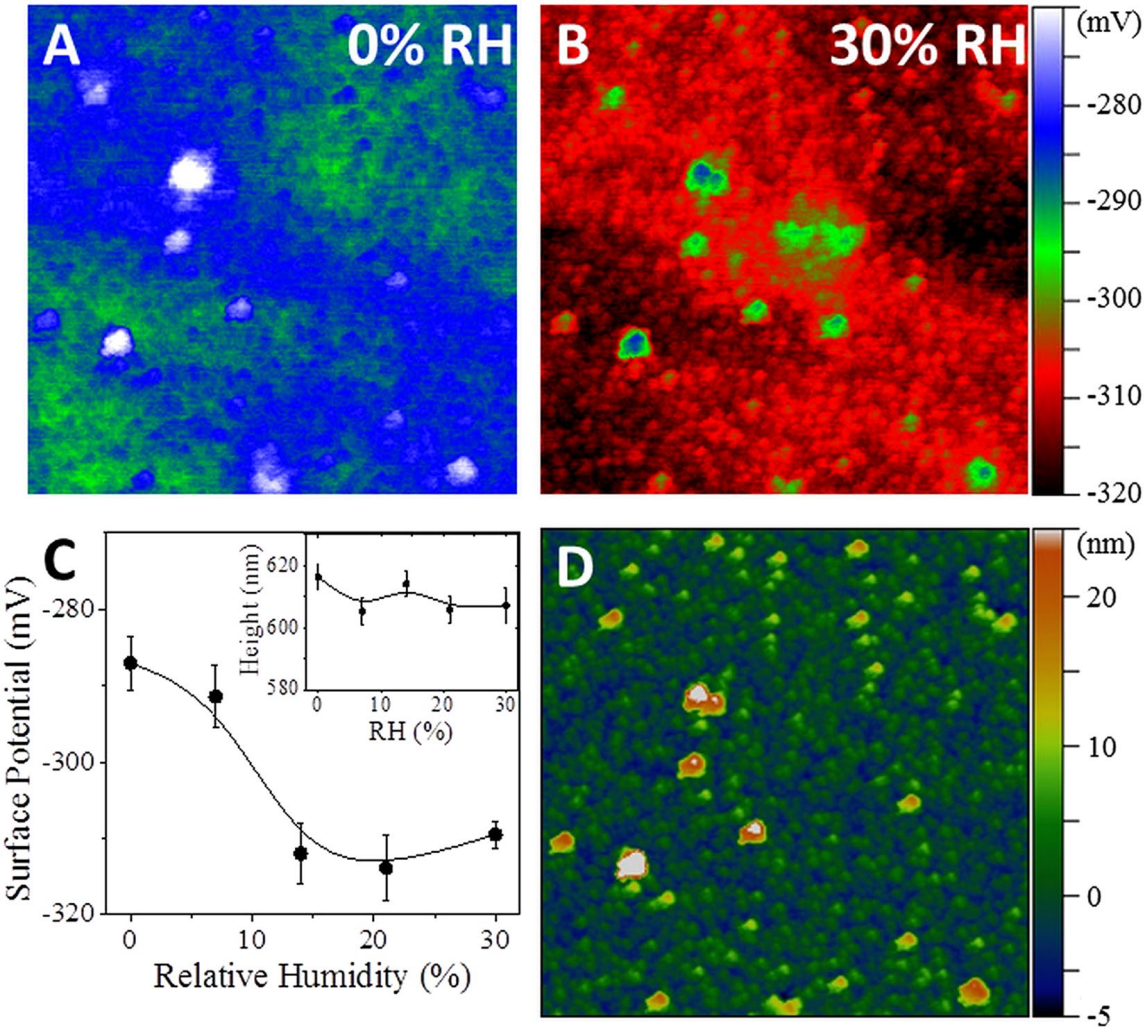
False color coded KPFM surface potential maps of a ZnO film on a polyimide substrate in a RH controlled Ar atmosphere at 0% RH (**A**) and 30% RH (**B**). Large aggregates are detached from the conductive network of crystallites, and thus do not show as much change in surface potential. Comparison of the averaged surface potential at each water concentration (**C**) shows the surface potential becoming increasingly negative with increasing humidity, which is expected from a reaction between water and the ZnO surface. Evidence of saturation above 14% likely corresponds with the formation of a water monolayer. No changes in the average AFM height (**C**, inset) to RH show that the measured surface potential response is *not* an artifact related to changes in film thickness. The topography of the ZnO surface is shown for reference (**D**).

To determine if water is adsorbed at oxygen vacancies or if it is hydrogen-bonded to the film surface, we used KPFM to monitor the surface potential of a single 5 µm^2^ area of the ZnO films as the relative humidity (RH) was increased^42–44^. The surface potential of the ZnO film was found to be dependent on the RH of the environment, Fig. 6A,B. The surface potential, averaged over the sample area, Fig. 6C decreased with relative humidity up to 14% RH, after which the surface potential plateaued, suggesting the changes observed until a monolayer is formed. The averaged AFM topography, Fig. 6C-inset, reveals that no significant height changes occurred in conjunction with the changes in RH. A few local areas showed much less of an effect to RH changes, which is likely due to small particles that are only loosely bound to the surface, and are not part of the continuous film. To verify that the surface potential changes were not due to changes in the work function of the measurement tip, relative changes between the loosely bound particles and the ZnO film was characterized, revealing a similar trend, Figure S4. The decrease in surface potential indicates that the ZnO surface becomes more electron rich with increasing RH, consistent with a change in surface functionality. This suggests that oxygen species, such as hydroxyl functionalities, may have replaced surface oxygen vacancies resulting in a more negative ZnO surface. Although the surface potential plateaued above 14% RH, the resistance and impedance properties continued to change at higher RH values. A more in-depth study of local changes to the electronic structure of metal oxide films in relation to the local environment is currently in progress to develop a better understanding of this phenomenon.

The resistive and capacitive response of ZnO depends on the specific interaction with the analyte. These interactions can be characterized by the energy of analyte adsorption, which is dependent on the surface termination (Zn or O), the density of Zn and O vacancies, and the presence of chemisorbed water molecules, i.e. the extent of surface hydroxylation. We used the Large-scale Atomic/Molecular Massively Parallel Simulator (LAMMPS) with a reactive force-field (ReaxFF) for Zn-O-H systems to estimate the effect of the vacancies in a ZnO (1010) surface on the adsorption of H_2_O and O_2_ molecules, with and without a monolayer of absorbed water^45^. A supercell consisting of 112 ZnO formula units (surface area 13.2 × 21.1 Å) was constructed and minimized in two dimensions using the Grand Canonical (GC) method with periodic boundary conditions. The third dimension was expanded to 55 Å and made non-periodic. ZnO surfaces with either zinc or oxygen atom vacancies were minimized in the same manner. For each type of surface we also considered a completely hydroxylated surface, with 100% of the adsorption sites having an adsorbed water molecule. Each calculation was done with the assumption that 2 underlying atomic layers were fixed.

The calculated absolute energy and defect energy values, as well as the resulting adsorption geometries are shown in Figure S5. Adsorption energies were calculated as the difference between the adsorbed configuration energy and the sum of the corresponding surface and molecule energies, summarized in Table S2. We find that the optimal adsorption geometry of a water molecule on an ideal ZnO surface closely matches previously reported geometries^46^. The resulting binding energy of such a configuration was found to be 1.11 eV, which was somewhat higher than the 0.94 eV previously found from density-functional theory calculations^38^.

Defect energies were calculated as the difference between total energy of the corresponding ideal surface and the energy of the surface with a vacancy. Oxygen defect formation is less favorable than zinc defect formation, but results in higher adsorption energy compared to the surfaces of the same type without defects or with zinc vacancies (Figure S5). The simulations show a different adsorption geometry of water molecules interacting with an oxygen vacancy lattice than with an ideal lattice, which corresponds more closely to the coordinate OH configuration described by Hu *et al*.^47^.

Hydroxylation of the surface results in a significant decrease in adsorption energy for both H_2_O and O_2_. On a clean surface, we observed an energy difference of 1.65 eV, depending on the defect, whereas the difference in adsorption energy between H_2_O and O_2_ on a hydroxylated surface (which more closely resembles a typical ZnO sensor surface) is only 0.78 eV, making experimental differentiation between the analytes difficult to determine. We also find that the adsorption energy on a surface with oxygen vacancies is 55% higher than on one without vacancies, which is in agreement with Hu *et al*.^47^. Adsorption of a single water molecule on a hydroxylated ZnO surface with an oxygen vacancy is 69% more energetically favorable than that on an ideal hydroxylated surface, which is in agreement with the 58% higher adsorption energy shown by Hu *et al*., and supports their conclusion that additional binding sites are created due to oxygen vacancies^47^. As previously discussed, the adsorption of either O_2_ or H_2_O on the ZnO surface will alter the electronic properties of the ZnO film, but the differences in adsorption energy between H_2_O and O_2_ result in resistive and capacitive signatures unique to each adsorbate.

As previously discussed, the migration of electrons to the surface due to water adsorption, explains the observed change in R_G_, while the change in surface functionalities could explain the observed changes in C_GB_. Thus, adsorption of water to the ZnO surface alters both the film resistance and the film capacitance. Furthermore, either resistive or capacitive measurements (below 40 kHz, or at 150 kHz, respectively) could be used for the measurement of O_2_ and H_2_O by ZnO films. However, differentiation between the two analytes is difficult directly from the resistance response. We argue that it is feasible to differentiate between the responses to H_2_O and O_2_ by using advanced statistical analysis algorithms.

**Figure 7:**
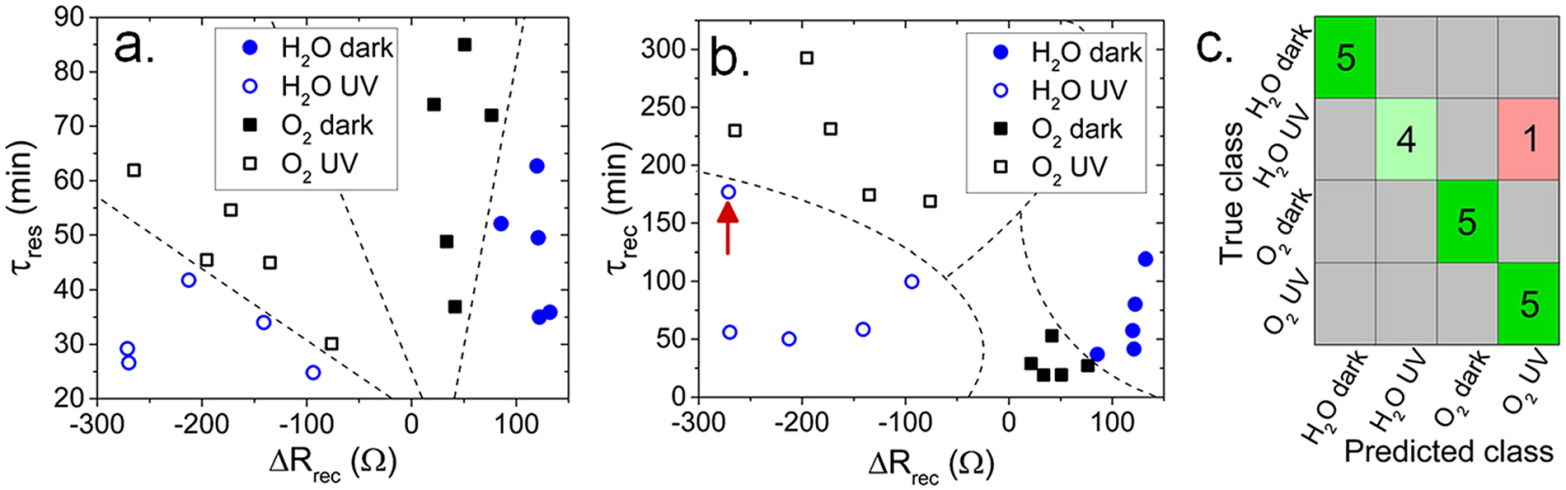
Example of LDC model used to distinguish between H_2_O and O_2_ environments in the dark and under UV irradiation in 2-dimensional τ_res_-ΔR_rec_ parameter space. (**a**) Example of environment classification using a CDC model in τ_rec_-ΔR_rec_ space. (**b**) Example of a confusion matrix for classification results using linear and cubic SVM learning. (**c**) The error corresponds to the point identified by the red arrow in (**b**).

Indeed, using machine learning and statistical analysis, we were able to distinguish between H_2_O and O_2_ analytes under dark and UV-irradiated conditions, even when the ZnO resistance does not reach steady-state conditions between the gas/vapor pulses. We extracted 4 predictor variables from the ZnO resistance response shown in Fig. 3: the resistance growth time constant during the response to gas/vapor (τ_res_), the resistance decay constant during recovery (τ_rec_), the resistance change in response to gas/vapor exposure (ΔR_res_), and the resistance change during recovery (ΔR_rec_). We defined 4 classes of environmental conditions: H_2_O in dark conditions (H_2_O_dark_), H_2_O under UV-irradiation (H_2_O_hν_), O_2_ under dark condition (O_2-dark_), and O_2_ under UV-irradiation (O_2-hν_). Using a support vector machine (SVM), a supervised machine-learning model with a quadratic kernel function implemented in the Matlab classification learning application, we demonstrated that is it possible to distinguish between the 4 classes of environmental conditions solely by analysis of the ZnO resistive response (Fig. 7a,b). Principal component analysis (PCA) was performed on the 4-dimensional predictor matrix to obtain optimized weights for initialization of the SVM. The optimized SVM correctly predicts the local environmental conditions at a ~95% confidence level. An example of SVM results is shown in the form of a confusion matrix in Fig. 7c. H_2_O_hν_ is the only condition which frequently produces a classification error for H_2_O and O_2_ exposure under UV-irradiation, primarily due to the similar values of τ_rec_ and ΔR_res_.

To investigate the performance of other classification techniques, we implemented a quadratic discriminant classifier (QDC) using diagonal covariance regularization in the Matlab classification learner application. Using only τ_res_ and ΔR_rec_, the QDC classifies 100% of the predictors without the need for PCA or other higher dimensional analysis. As shown in Fig. 5B, the ZnO responses to all 4 conditions (H_2_O_dark_, H_2_O_hν_, O_2-dark_, and O_2-hν_) can be distinguished by QDCs, enabling identification of H_2_O/O_2_ and detection of UV light. Characterization of the ZnO response by the QDC is less computationally-intensive than analysis by the SVM because it does not require data preprocessing using PCA, and it omits τ_rec_ and ΔR_res_ inputs, which confines predictor variables to a 2-dimensional parameter space. The vertical arrow in Fig. 7b identifies the point which was classified incorrectly by the SVM. It is likely that the error is avoided by the QDC because lower-dimensional parameter space minimizes the probability of overfitting. The results shown here demonstrate that even in the absence of steady-state, and reversible electronic responses, ZnO resistance changes can be used for distinguishing between H_2_O and O_2_ in the dark and under UV irradiation.

## Conclusions

We have shown that the changes in the electrical properties of ZnO devices fabricated from low-cost, biologically-derived nanoparticles can be used to effectively monitor environmental changes in O_2_ and H_2_O. The distinct adsorption energies between O_2_ and H_2_O on polycrystalline ZnO indicate that these adsorbates interact with the ZnO surface through distinct mechanisms. Under UV irradiation O_2_ reacts with photogenerated electrons resulting in chemisorption of ionic oxygen to the ZnO surface. On the other hand, H_2_O reacts with both photogenerated holes and electrons from the depletion layer, resulting in dissociation and thus alteration of the surface polarization, ultimately resulting in a larger impedance response, as compared to O_2_. Grain resistance and grain boundary capacitance of polycrystalline ZnO film dominate the response of both O_2_ and H_2_O in dark and under UV irradiation, while response from the grain boundary resistance and grain capacitance are only significant under UV irradiation. By using statistical and machine learning approaches we have demonstrated that electrical signals (steady state and dynamic sensor response and recovery) can be used to discriminate between the analytes, as well as the dark/UV irradiated conditions under which the measurements were made. MD simulation of polycrystalline ZnO films show that Zn and O vacancies have deterministic effects on the energy of adsorption of O_2_ and H_2_O, and thus allow for further tunability of the sensor response.

## Methods

Bacterial-derived ZnS nanoparticles were synthesized through biological nanofermention by abiotic *Thermoanaerobacter* sp. X513, using previously reported methods^48–50^. The ZnS nanoparticles, Figure S6, were thermally oxidized at 800 °C for 5 hours under continuous flow of oxygen at 300 SCCM, and the complete oxidation of ZnS to ZnO was verified using X-ray diffraction (XRD) and Energy-dispersive X-ray spectroscopy (EDS), showing no detectible residual ZnS. ICP analysis was performed on the bacterially-synthesized ZnS and the oxidized ZnO to verify purity of the samples, Table S3, and indicate that no detectable contaminates are present from the bacterial growth media or through the oxidation process. After oxidation, the ZnO was pressed into a ¾” pellet by uniaxial cold-pressing at 7 tons, and this pellet was used to prepare ZnO films by pulsed laser deposition (PLD).

Pulsed-laser deposition (PLD) of ZnO films can result in film morphologies ranging from nanoparticles or nanowires to thin films, depending on the PLD conditions, such as background pressure and substrate temperatures^51, 52^. We focused on ZnO films synthesized in conditions suitable for flexible substrates (250 °C and 5 × 10^−6^ Torr background pressure)^52, 53^. Prior to growth, polyimide (Kapton) substrates (2 cm × 2 cm) were consecutively rinsed and sonicating in water, isopropanol, and acetone for 15 min each, and then dried under N_2_ gas. The substrates were further cleaned for 5 minutes in an UV Cleaner (No. 42, Jelight Company Inc., Irvine, CA), and then affixed to a temperature controlled heater, set to 250 °C in a PLD system. The target-to-substrate distance was fixed at 5 cm. The ZnO films were deposited in conditions suitable for flexible substrates and high conductivity, 250 °C and 5 × 10^−6^ Torr background O_2_ pressure and consisted of 5000 pulses from a 248 nm excimer laser, operated at 1 Hz. The spot size of the laser on the ZnO target was 1.8 mm^2^, resulting in a fluence of 3.5 J/cm^2^. The target was rotated continuously at 10 RPM to avoid target pitting.

Structural characterization of the films was performed using a ZEISS Merlin Scanning Electron Microscopy (SEM) and a PANalytical powder diffractometer (XRD). The ZnO films were found to be highly textured, columnar structures with the hexagonal crystal basal plane parallel to the substrate surface. Analysis of the XRD data indicated the average crystallite size ranged from 5–25 nm and the film thickness was measured to be 85 nm by SEM, as shown in Fig. 2. Elemental composition of the film was characterized by energy-dispersive X-ray spectroscopy, and is solely composed to Zn and O, Figure S7, with C, Al, and Si signals originating from the carbon tape and the sample stage. Surface potential measurements were performed by Kelvin Probe Force Microscopy (KPFM) using a commercial Atomic Force Microscope (AFM) – i.e., a Cypher ES (Asylum Research an Oxford Instruments Company).

Electrical characterization of the films was performed after electron beam deposition of electrodes (5 nm Ti and 50 nm Au) on the ZnO surface. The resulting electrodes were 1 mm × 5 mm, with a gap of approximately 0.5 mm between them. Silver paste was used to affix wires to the electrodes. Resistance and impedance measurements were recorded using a Keithley 2420 SourceMeter, and Zahner IM6 impedance analyzer, respectively. The measurements were conducted in a special environmental system that was co-developed with Surface Measurement Systems (London, UK), hereafter referred to as the environmental chamber. The measured electrical properties between the two surface electrodes were dominated by in-plane charge transport, and thus the electrical properties were modeled as the sum of the electrical properties of the grain and grain boundaries of the ZnO crystallites.

The high binding energy between ZnO and O_2_/H_2_O is expected to limit desorption, so UV light from an 8 Watt mercury lamp was used to facilitate desorption and recovery. UV irradiation of the ZnO film resulted in an exponential decrease in film resistance, likely due to the photocatalytic removal of surface adsorbates, including oxygen, Figure S1 ^54^. Prior to any environmental response measurements, the ZnO films were first UV-activated for 24 hours (for resistance stabilization) by UV irradiation from an 8 W mercury lamp in a vacuum of 5 × 10^−6^ Torr.

## Electronic supplementary material

Supplementary Information
