# Supplementary material for: UV-activated ZnO films on a flexible substrate for room temperature O2 and H2O sensing
Source: Sci Rep. 2017 Jul 20;7:6053. doi: 10.1038/s41598-017-05265-5 (PMC5519692; doi:10.1038/s41598-017-05265-5)
Supplement: Supplementary file 1 — Supplementary Information [file 41598_2017_5265_MOESM1_ESM.pdf]

SUPPLEMENTARY INFORMATION

UV-activated ZnO films on a flexible substrate for room temperature O_2 and H_2O sensing

*Christopher B. Jacobs[§], Artem B. Maksov[§†], Eric S. Muckley[§†], Liam Collins[§], Masoud Mahjouri-Samani[§], Anton Ievlev[§], Christopher M. Rouleau[§], Ji-Won Moon[#], David E. Graham[#], Bobby G. Sumpter[§,‡], and Ilia N. Ivanov[§]**

[§] Center for Nanophase Materials Science and Institute for Functional Imaging of Materials, Oak Ridge National Laboratory, PO Box 2008, Oak Ridge TN, USA
[†] The Bredesen Center for Interdisciplinary Research and Graduate Education, 444 Greve Hall, 821 Volunteer Boulevard, Knoxville, Tennessee 37996–3394, United States
[#] Microbial Ecology & Physiology Group, Biosciences Division, Oak Ridge National Laboratory (ORNL), PO Box 2008, Oak Ridge TN, USA
[‡] Computer Science & Mathematics Division, Oak Ridge National Laboratory, PO Box 2008, Oak Ridge, TN USA

TABLE OF CONTENTS

Figure S1	Stabilization of UV-induced resistance decrease during the UV-activation period prior to adsorption measurements.	S-2
Table S1	Table S1. Response and recovery times of resistance changes in response to O_2 and H_2O pulses	S-3
Figure S2	Resistive response to consecutive pulses of O_2 or H_2O vapor under UV conditions	S-4
Figure S3	Impedance spectra of ZnO films measured during oxygen and H_2O pulse experiments under UV irradiation.	S-5
Figure S4	Comparison of the relative surface potential difference as a function of relative humidity	S-6
Figure S5	Optimized structure and energy of different ZnO terminations including Zn and oxygen vacancies, calculated using LAMMPS molecular dynamics simulator with ReaxFF potential..	S-7
Table S2	Calculated differential energies (eV) of adsorption of water and oxygen on clean and hydroxylated surface of ZnO referenced to the energy of surface and single molecule.	S-8
Figure S6	TEM of bacterial-synthesized ZnS nanoparticles	S-9
Table S3	Elemental analysis by inductively coupled plasma mass spectrometry	S-10

Figure S7 Compositional Elemental analysis of ZnO film deposited by PLD S-11

RESULTS

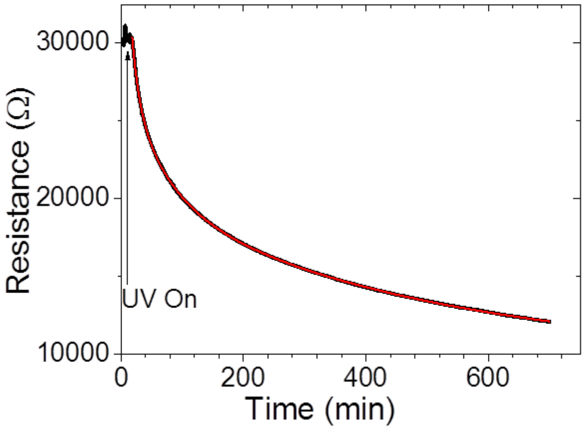

Figure S1. Resistance response to UV irradiation (A) shows a complex decay that stabilizes over time to a constant linear drift. The ZnO film was allowed to stabilize for > 24 hours prior to adsorption measurements.

Table S1. Response and recovery times of resistance changes in response to O_2 and H_2O pulsess

Adsorption Period					
Δ Pressure O_2 (Torr)	2.7	8.0	13.4	18.7	21.5
Dark (min^{-1})	72.0 ± 2.0	85.0 ± 2.6	36.9 ± 0.6	48.8 ± 1	74.0 ± 3.0
UV (min^{-1})	30.1 ± 0.2	45.0 ± 0.2	54.6 ± 0.4	45.5 ± 0.3	61.9 ± 0.6
Δ Pressure H_2O (Torr)	2.7	8.0	13.4	18.7	21.5
Dark (min^{-1})	52.1 ± 0.4	35.0 ± 0.3	62.7 ± 0.8	49.5 ± 0.5	35.9 ± 0.3
UV (min^{-1})	24.8 ± 0.1	34.0 ± 0.2	41.8 ± 0.4	26.6 ± 0.1	29.2 ± 0.2
Desorption Period					
Δ Pressure O_2 (Torr)	-2.7	-8.0	-13.4	-18.7	-21.5
Dark (min^{-1})	27.1 ± 0.6	19.2 ± 0.5	53.0 ± 3.0	19.0 ± 0.6	29.0 ± 0.5
UV (min^{-1})	168.7 ± 17.1	174.3 ± 9.0	231.4 ± 6.0	293 ± 18.4	229.9 ± 6.2
Δ Pressure H_2O (Torr)	-2.7	-8.0	-13.4	-18.7	-21.5
Dark (min^{-1})	37.0 ± 0.8	80.2 ± 10.3	57.5 ± 2.4	41.6 ± 1.4	119.1 ± 0.9
UV (min^{-1})	99.7 ± 5.2	58.5 ± 1.0	50.2 ± 1.2	56.0 ± 4	176.8 ± 0.9

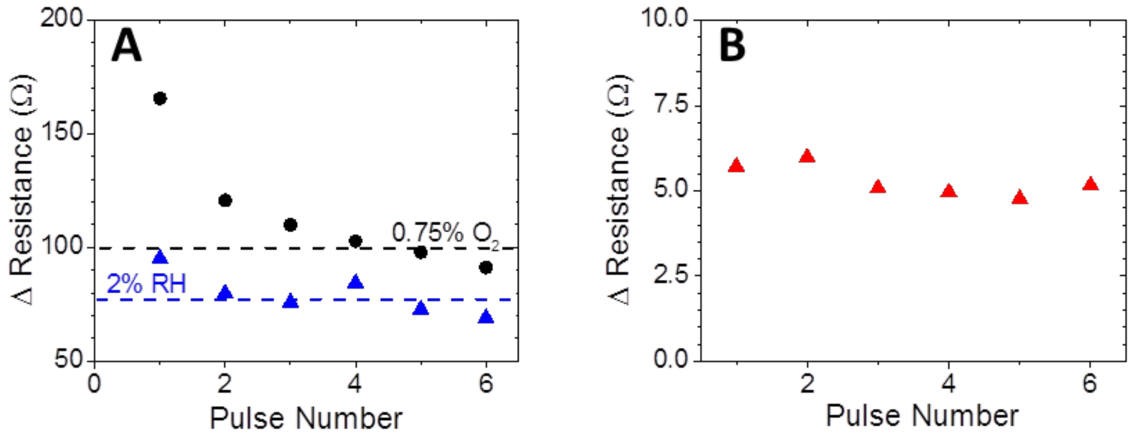

Figure S2. Resistive response to six consecutive 2 minutes pulses of O_2 or H_2O vapor (0.75% or 2%, respectively) under UV conditions (A) shows a decreasing resistive response to each subsequent pulse of equal concentrations, whereas no change in response is observed from consecutive pulses of Ar gas (B). The disparity in responses to H_2O and O_2 trends suggests that the ZnO may be deoxygenated by UV irradiation in vacuum conditions, and that that the response of oxygen or water are related due to adsorption, and not to piezoelectric effects from the pressure change.

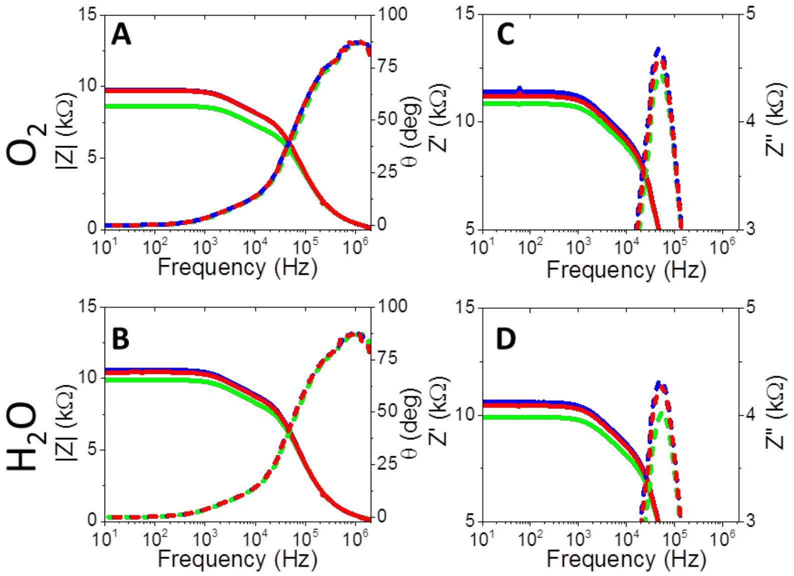

Figure S3. Impedance spectra represented as Bode plots of ZnO films measured during oxygen (A), and H_2O (B) pulse experiments under UV irradiation. Solid traces represent complex impedance and dashed lines show phase. The green trace was measured during the initial vacuum, blue trace during the first pulse (1.7% O_2 (A) or 11.25% H_2O), and red trace during the recovery vacuum after the pulse. The corresponding colored arrows in Figure 2 illustrate the time that each trace was measured. Plots of real (solid) and imaginary (dashed) impedance vs frequency curves (C,D) show resistive dominating behavior at frequencies below 40 kHz, and maximum capacitive behavior at 150 kHz. Both resistance and capacitance increase with exposure to oxygen or water, and decrease during the vacuum recovery period.

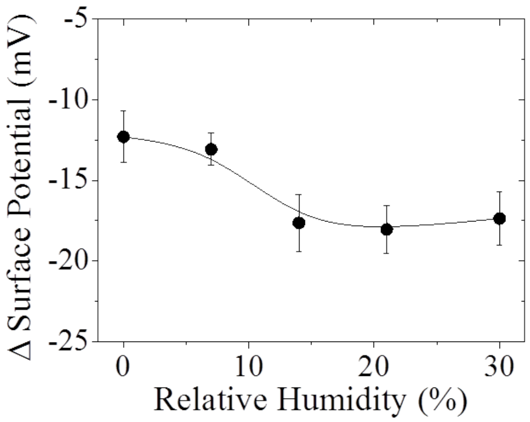

Figure S4. Comparison of the relative surface potential difference between the ZnO surface and loosely attached particles, at each water concentration, shows that the surface potential becomes increasingly negative with increasing humidity, but plateaus beyond 14% RH.

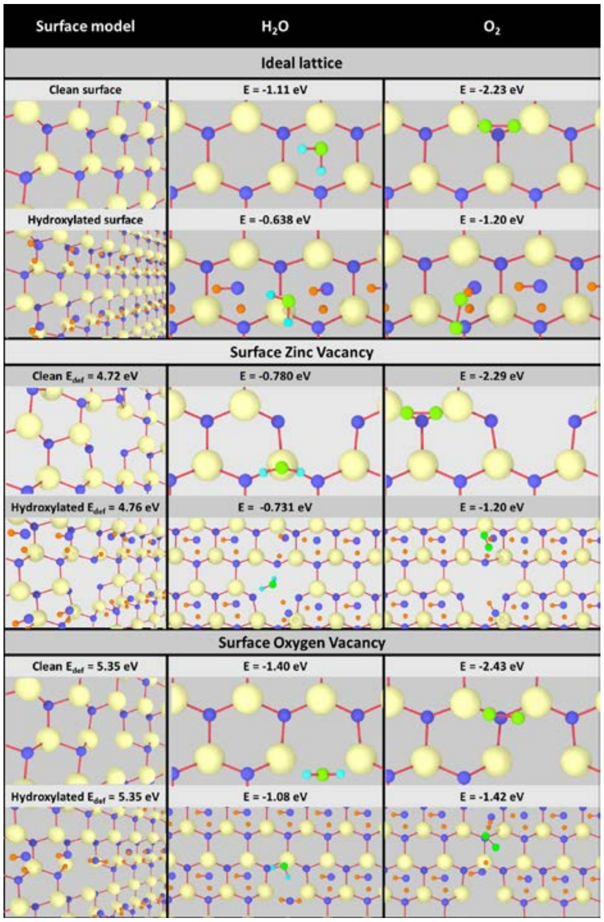

Figure S5. Optimized structure and energy of different ZnO terminations including Zn and oxygen vacancies were calculated using LAMMPS molecular dynamics simulator with ReaxFF potential. A supercell consisting of 112 ZnO formula units (surface area 11.4 x 21.1 Å) was constructed and minimized using CG method with periodic boundary conditions in two dimensions, and a third expanded to 55 Å and made non-periodic. The energy of water and oxygen adsorption for ideal and non-ideal ZnO surfaces was calculated. The adsorption energy of oxygen and water are significantly lower for the hydroxylated surface. The latter was obtained by minimizing the energy of a monolayer of water molecules on ZnO surface. Simulations indicate that water exists in a pre-dissociative state. Total energies of the minimum configuration of ZnO for different cases of surface termination are shown in Table S2.

Table S2. Calculated differential energies (eV) of adsorption of water and oxygen on clean and hydroxylated surface of ZnO referenced to the energy of surface and single molecule.

**Model**		**H_2O, eV**	**O_2, eV**
**Ideal lattice**	Clean	-1.13	-2.23
	Hydroxylated	-0.64	-1.20
**Zinc vacancy**	Clean	-0.78	-2.28
	Hydroxylated	-0.73	-1.20
**Oxygen Vacancy**	Clean	-1.40	-2.43
	Hydroxylated	-1.08	-1.41

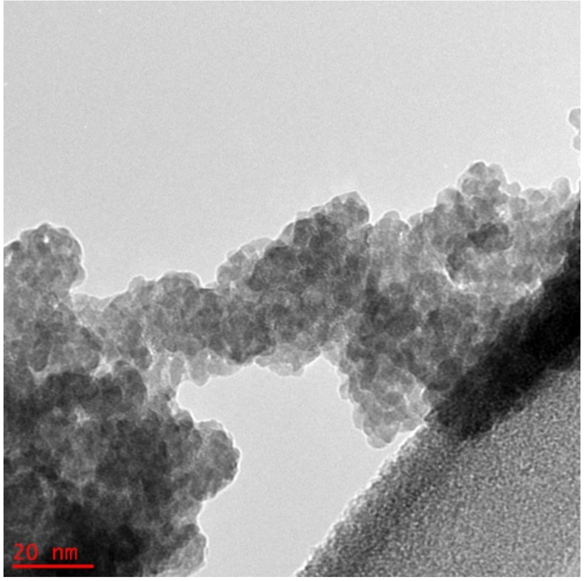

Figure S6. TEM image of bacterially-synthesized ZnS nanoparticles indicate the diameters of the nanoparticles are 4-5 nm.

Table S3. Elemental analysis by inductively coupled plasma mass spectrometry

	Detection Limit (ppm)	ZnS (ppm)	ZnO (ppm)
**Fe**	8	84	95
**Ni**	10	22.5	
**Cd**	7	< 6.9	< 7.2
**Pb**	7	< 6.9	< 7.2
**As**	3	< 2.8	< 2.9
**Co**	8	< 7.9	
**Cu**	8	< 7.9	
**Mn**	10	11.7	

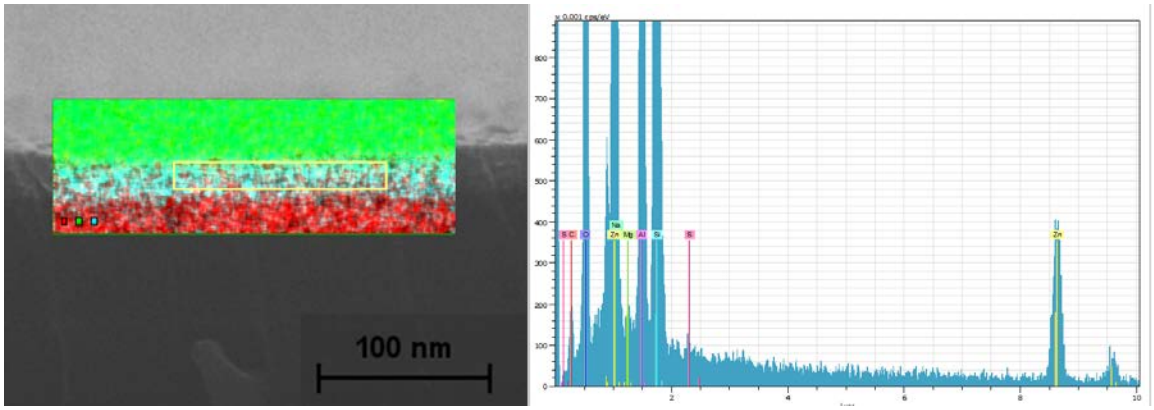

Figure S7. Analysis of deposited ZnO film composition by energy-dispersive X-ray spectroscopy indicates the film is primarily composed of zinc and oxygen, with no sulfur, iron, or nickel distinguishable from the background. Carbon, Silicon, and Al peaks can be attributed to carbon tape, the Si substrate, and the aluminum sample stage, respectively.
